# Comparative study on hemoglobin A1c, glycated albumin and glycosylated serum protein in aplastic anemia patients with Type 2 diabetes mellitus

**DOI:** 10.1042/BSR20192300

**Published:** 2020-05-22

**Authors:** Minghuan Suo, Dongmei Wen, Weijia Wang, Tingting Zhang

**Affiliations:** Laboratory Medicine Centre, Zhongshan Hospital Affiliated to Sun Yat-sen University, Zhongshan 528403, Guangdong, China

**Keywords:** aplastic anemia, glycated albumin, glycated serum protein, glycosylated hemoglobin

## Abstract

**Objective:** To differentiate the value of hemoglobin A1c (HbA1c), glycated albumin (GA) and glycosylated serum protein (GSP) in monitoring blood glucose of patients with aplastic anemia.

**Methods:** 42 patients with aplastic anemia (AA) and 30 patients with AA and Type 2 diabetes mellitus (T2DM) were enrolled in the study, in comparison with 114 healthy subjects and 88 subjects with T2DM. HbA1c, GA, GSP, fasting plasma glucose (FPG), hemoglobin (Hb) and albumin (ALB) were measured, and group comparison and correlation analysis were carried out.

**Results:** Compared with the non-diabetes patients while ALB were <30 g/l or 30–40 g/l, the HbA1c and GSP values in AA, T2DM and AA+T2DM patients were significantly higher while the GA values were lower. Moreover, no differences in FPG levels. The AA+T2DM patients with ALB >40 g/l had higher HbA1c level, with no difference in GA, GSP and FPG levels. There was a positive correlation between HbA1c and GA in healthy group (ALB ≥ 40 g/l), AA patients (ALB 30–40 g/l and ≥40 g/l), T2DM patients (ALB 30–40 g/l and ≥40 g/l) and AA+T2DM patients (ALB 30–40 g/l and ≥40 g/l) but not in those with ALB < 30 g/l.

**Conclusion:** The HbA1c results were affected by moderate-to-severe anemia, but not mild anemia. HbA1c is not recommended to detect blood glucose levels in AA patients (Hb < 90 g/l) or AA patients (ALB < 30 g/l). FPG and GSP are not suitable for AA patients.

## Introduction

Since the 1990s, World Health Organization (WHO) and the diabetes associations or societies in many countries have recommended HbA1c as the preferred diagnostic index for monitoring diabetes but more recently has also been advocated as a diagnostic tool for T2DM [1–5], while HbA1c is also generally recognized as the “gold standard” for blood glucose testing. However, HbA1c has some limitations. Several studies have shown that HbA1c cannot be used to accurately assess blood glucose levels under certain circumstances, such as changes in red blood cell life and imbalance in the proportion of young and mature erythrocytes [6,7], Hb metabolic disorders and the use of erythropoietin [8,9].

Glycated serum protein (GSP) is a product of non-enzymatic reaction between blood glucose and plasma protein (approximately 70% of which is albumin). The determination of glycosylated serum protein (GSP) is also called fructosamine determination. Glycosylated serum protein (GSP) measurement reflects the total glycosylated plasma protein in plasma, its value is susceptible to the influence of protein concentration, bilirubin, chyle and low molecular weight substances in blood, especially in patients with hypoproteinemia and abnormal albumin transformation. At the same time, non-specific reducing substances in serum can also react with glycation sites. The specificity of glycosylated serum protein (GSP) assay is poor because of the different reaction rates.

GA is an emerging indicator for blood glucose monitoring; several studies have suggested that GA is more suitable in patients with certain diseases, such as hemolytic anemia, hepatic cirrhosis with hyperglycemia, than HbA1c [10,11]. GA is the product of glucose and serum albumin in non-enzymatic reactions, representing the average level of blood glucose in recent 2–3 weeks. GA relative to HbA1c can better reflect the changes or fluctuations in blood glucose level. In addition, several investigators have suggested that, compared with HbA1c, GA is more suitable as a diagnostic parameter for recessive diabetes and stress hyperglycemia [12] and as a monitoring glycemic control in patients with anemia [13]. Although there are many advantages of GA over HbA1c, it also has some limitations that it could be affected by changes in the structure and half-life of albumin [14].

In patients with aplastic anemia, the red blood cell life and hemoglobin metabolism are affected by their abnormal proliferation of bone marrow. Therefore, it is particularly important to develop and screening of diabetes and monitoring of glycemic control status for patients with aplastic anemia and those with diabetes. At present, there is no report on comparative studies of the application value of blood glucose monitoring indexes in patients with aplastic anemia in China. The present study was designed to compare the results of HbA1c, GA and GSP detected in patients with aplastic anemia and to explore their clinical value as monitoring indicators for blood glucose in patients with AA and AA+T2DM. It was hoped that our results will help select adequate blood glucose monitoring indicators for those patients in clinical practice.

## Materials and methods

### Materials

This cross-sectional study began in 2012; we enrolled consecutive patients with AA and AA+T2DM seen in Zhongshan Hospital, Zhongshan University (Zhongshan, Guangdong, China) into the study, after an informed consent was signed by each patient. There were four groups in the present study: AA group, including 42 patients with aplastic anemia (20 males and 22 females), aged (51 ± 17) years; AA+T2DM group, including 30 patients (12 males and 18 females), aged (58 ± 19) years; healthy control group, including 114 healthy volunteers (62 males and 52 females), aged (51 ± 17) years; 88 T2DM patients were involved (47 males and 41 females), aged (57 ± 15) years. There were no statistically significant differences in age and gender distributions among the four groups in the present study.

### Inclusion and exclusion criteria

#### AA group

Patients diagnosed of AA, according to the AA diagnostic criteria set forth by the Fourth National Conference on Aplastic Anemia of China (1987): (1) Whole blood cell decreased, the absolute value of reticulocytes decreased; (2) Generally no splenomegaly; (3) Bone marrow examination showed at least one site of hyperplasia or severe reduction (such as hyperplasia active, megakaryocytes should be significantly reduced, bone marrow granule components should see non-hematopoietic cells increase. Bone marrow biopsy and other examinations should be performed); (4) Excluded from other diseases that could have pancytopenia, such as paroxysmal nocturnal hemoglobinuria, myelodysplastic syndrome in patients with refractory anemia, acute hematopoietic dysfunction, bone marrow fibrosis, acute leukemia and malignant histocytosis; (5) No response to anti-anemia drug treatment; no thyroid diseases, liver cirrhosis and nephrosis.

#### AA+T2DM group

AA patients who also met the 2010 WHO Diagnostic Criteria for Diabetes: FPG ≥ 7.0 mmou/l and/or HbA1c ≥ 6.5%. For both AA and AA+T2DM groups, patients with thyroid disease, liver disease, chronic kidney disease and other diseases that may interfere with the laboratory testing were excluded. In the present study, the lower limit of hemoglobin of 110 g/l was used for diagnosis of anemia, and albumin <30 g/l was used for diagnosis of hypo-albuminemia [15].

#### T2DM group

Patients diagnosed of T2DM according to the 2010 WHO Diagnostic Criteria for Diabetes: FPG ≥ 7.0 mmou/l and/or HbA1c ≥ 6.5%. T2DM patients with anemia, hypoalbuminemia, thyroid gland disorders, liver diseases, chronic kidney disease and other chronic diseases were excluded. Serum creatinine <1.2 mg/dl was used as an indicator of normal renal function.

#### Healthy control group

The healthy control samples were screened using criteria provided by the Physical Examination Center in Affiliated Zhongshan Hospital Affiliated to Sun Yat-sen University, described briefly as follows: no chronic disease (diabetes, hypertension, hyperlipidemia, gout and chronic kidney disease) or regular long-term medication; no liver diseases, not HBV or HCV carriers; no thyroid diseases.

### Chemicals, reagents and instrumentation

The reagents and instruments used in the present study were as follows: (1) HbA1c detection was accomplished using Purimus Ultra2 Hemoglobin A1c analyzer that was based on the principle of high performance liquid chromatography (HPLC); the results were compared with that using Variant Ⅱ Turbo hemoglobin analyzer (Bio-Rad) and there was no statistically significant difference between the two methods (*P* = 0.783). (2) GA analysis was performed on Siemens ADVIA 2400 automatic biochemical analyzer, using the bromocresol purple method with the Lucica GA-L glycated albumin kit, Asahi Kasei Pharmaceutical Co., Ltd (Japan; the reference range, 11–16%). (3) ALB determination was performed with Lucica GA-ALB glycated albumin kit from the GA testing system. (4) Hb was analyzed on the XE-2100 automatic blood cell analyzer, using the RF/DC detection method. (5) GSP was analyzed on the Siemens ADVIA 2400 automatic biochemical analyzer, using the colorimetric method.

### Statistical analysis

The data and statistical analyses were performed with the SPSS19.0 (SPSS, Chicago, Illinois). The measurement data were expressed as mean ± SD. The comparisons among different groups were accomplished using ANOVA or Student’s *T* test, as appropriate. The correlation analyses were conducted using Pearson correlation analysis and linear regression analysis. All *P* values are two-tailed, *P* < 0.05 and *P* < 0.001 were considered statistically significant or extremely significant, respectively.

## Results

### Correlation analysis of FPG levels during the study period of two months

We first analyzed the correlations among the FPG levels at three time points (0, 1 and 2 months) in the AA and AA+T2D groups, using Pearson correlation analysis. The correlation coefficients were as follows: for the AA group, *r* = 0.678 (*P* = 0.000), *r* = 0.794 (*P* = 0.000) and *r* = 0.861 (*P* = 0.000), respectively; for the AA+T2DM group, *r* = 0.856 (*P* = 0.000), *r* = 0.866 (*P* = 0.000) and *r* = 0.970 (*P* = 0.000), respectively. The results indicated that the blood glucose levels were high correlation in the two groups of patients.

### Comparison of mean parameters among different groups

As shown in Table 1, the mean HbA1c level of the AA group was significantly higher than that of the healthy control group (*P* < 0.05). The mean GSP level of the AA group was also significantly higher than that of the healthy control group (*P* < 0.05).

**Table 1:** Comparison of monitoring indexes of blood glucose concentration between the experimental group and the control group (mean ± SD)

Group	Proportions	HbA1c (%)	HbA1c	GA (%)	GSP (μmol/l)	2Hpg (mmol/l)
Healthy control group	114	5.22 ± 0.42*	33.54 ± 4.56	12.60 ± 1.46	171.91 ± 27.97*	5.21 ± 0.54
AA	42	5.48 ± 0.49*	36.34 ± 5.31	12.46 ± 2.03	185.55 ± 28.90*	5.11 ± 0.50
T2DM	88	7.49 ± 1.25^A^	58.32 ± 13.71	21.65 ± 4.54	257.53 ± 53.82*	9.67 ± 3.72
AA+T2DM	30	8.45 ± 1.47^A^	66.89 ± 16.05	21.97 ± 3.58	283.81 ± 48.28*	9.98 ± 4.43

* *P*<0.05; ^A^*P*<0.001.

However, there were no statistically significant differences in the mean GA and 2hPG levels between the AA group and the healthy control group. The mean HbA1c level in the AA+T2DM group was significantly higher than that in the T2DM group (*P* < 0.001). The mean GSP level of the AA+T2DM was also significantly higher than that in the T2DM group (*P* < 0.05). There were no significant differences in the mean of GA and 2Hpg levels between the two groups (Table 1).

### Comparison of confounding factors among different groups

Several factors such as anemia, hypoalbuminemia, hyperlipidemia and hyperbilirubinemia may affect the results of HbA1c, GA, GSP and FPG. Therefore, these confounding factors should be controlled in the present study. As shown in Table 2, the mean levels of hemoglobin and albumin in the AA and AA+T2DM groups were significantly lower than that in the healthy control group (*P* < 0.05). The mean levels of total bilirubin (TBIL) and indirect bilirubin (IBIL) in the AA+T2DM groups were significantly lower than that in the T2DM group (*P* < 0.001). Of note, although the TBIL and IBIL levels were significantly different between the two groups, they were still within the normal reference range, indicting there was not liver dysfunction in either group.

**Table 2:** Comparisons of laboratory results among the four groups (mean ± SD)

Parameter	Healthy group versus AA group		T2DM group versus AA+T2DM group	
	Healthy control	AA	T2DM	AA+T2DM
HB (g/l)	139.67 ± 13.27*	81.07 ± 24.41*	136.67 ± 16.53*	83.93 ± 22.69*
ALB (g/l)	42.44 ± 3.76^A^	39.66 ± 5.92^A^	41.49 ± 5.07*	35.74 ± 6.68*
TBIL (μmol/l)	11.20 ± 4.22	9.33 ± 4.92	12.00 ± 4.97^A^	7.91 ± 4.54^A^
DBIL (μmol/l)	4.14 ± 2.06	3.59 ± 2.42	4.41 ± 2.89	3.28 ± 1.98
IBIL (μmol/l)	7.06 ± 3.62	5.83 ± 3.54	8.24 ± 4.61^A^	4.43 ± 3.38^A^
Urea (mmol/l)	4.94 ± 1.43	6.85 ± 3.84	5.42 ± 2.18	6.70 ± 3.94
Cr (μmol/l)	66.93 ± 16.80	71.97 ± 22.54	73.39 ± 27.99	74.10 ± 29.65
UA (μmol/l)	295.49 ± 54.90	291.33 ± 75.91	294.58 ± 77.02	290.00 ± 76.68
TC (mmol/l)	4.47 ± 0.76	4.72 ± 1.68	4.87 ± 1.09	4.30 ± 1.01
TG (mmol/l)	1.00 ± 0.46	1.42 ± 0.56	1.41 ± 0.55	1.44 ± 0.40

* *P*<0.05; ^A^*P*<0.001.

### Comparisons of blood glucose parameters in the AA group classified by the Hb level

**Figure 1:**
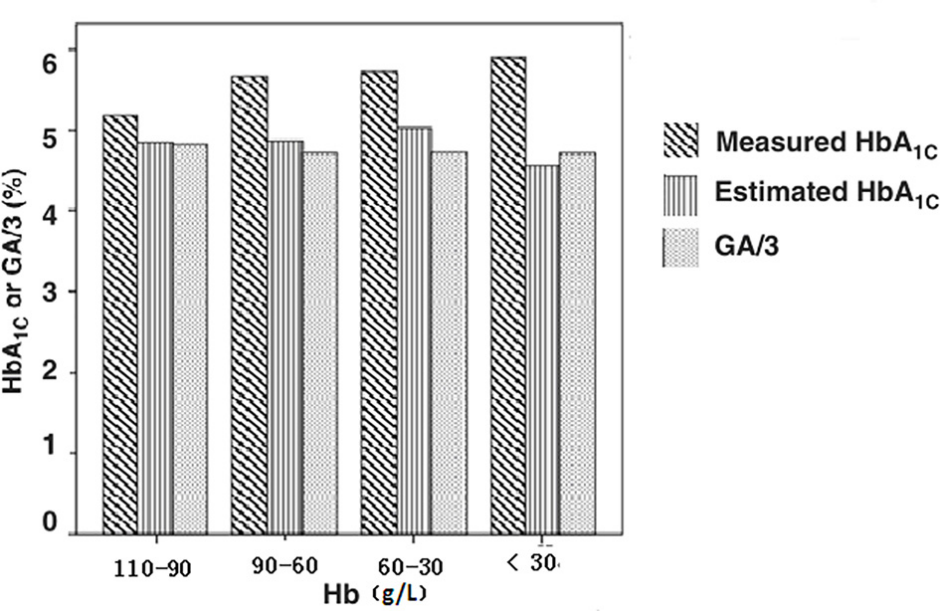
Comparison between estimated HbA 1C and measured HbA 1C or GA/3 in the AA group classified by the Hb level

To understand the effects of the Hb level on the blood glucose parameters in the AA group, we divided the AA patients into five subgroups according to their Hb levels and the results are shown in Table 3. Compared with the health control group, the AA patients with Hb >110 g/l group and Hb 90–110 g/l showed no changes in HbA1c levels, while there were significantly higher in other subgroups (*P* < 0.05). There was no strict control of albumin levels in the present study, the comparisons of GA levels may not be meaningful. The mean level of GSP in the Hb 90–110 g/l group was significantly higher that the healthy group (*P* < 0.001). There was no significant difference in the mean FPG levels among the groups. With the increase in anemia, the HbA1c value was significantly higher than the HbA1c estimates, which were based on the formula conversion [16], while GA / 3 value was very close to the HbA1c estimates (Figure 1).

**Table 3:** Comparisons of blood glucose parameters in AA group classified by the Hb level (means ± SD)

Group (g/l)	*n*	HbA1c (%)	GA (%)	GSP (μmol/l)	FPG (mmol/l)
Healthy control (Hb >110)	114	5.22 ± 0.42	12.60 ± 1.46	171.91 ± 27.97	5.10 ± 0.53
AA group					
Hb <30	3	6.14 ± 0.52*	14.86 ± 1.55*	174.60 ± 6.40	4.64 ± 0.10
Hb 30–60	5	5.72 ± 0.71*	14.06 ± 2.35*	183.84 ± 13.83	5.20 ± 0.66
Hb 60–90	19	5.69 ± 0.43*	12.31 ± 2.03	182.79 ± 33.12	5.02 ± 0.51
Hb 90–110	10	5.30 ± 0.39	12.61 ± 1.40	194.55 ± 12.01^A^	4.98 ± 0.40
Hb >110	5	5.07 ± 0.2	10.84 ± 2.14	182.90 ± 22.36	4.93 ± 0.77

* *P*<0.01; ^A^*P*<0.001.

### Comparisons of blood glucose parameters in the AA group classified by the albumin level

According to the levels of albumin, the AA group and the T2DM group were divided into two subgroups and the AA+T2DM group into three subgroups. As shown in Table 4, the GA level of the albumin 30–40 g/l group was significantly lower than that of the healthy group (*P* < 0.05), while the HbA1c value was also significantly higher than that of healthy group (*P* < 0.05). The mean GSP value of the albumin >40 g/l group was significantly higher than that of the healthy group (*P* < 0.001). The mean level of HbA1c in the T2DM group was significantly lower than that in the AA+ T2DM group (*P* < 0.05). In the T2DM group, the ALB 30–40 g/l subgroup and the ALB ≥ 40 g/l (*P* < 0.05). The GA level of the AA+T2DM with albumin <30 g/l group was significantly lower than that of T2DM group (*P* < 0.05). The mean GSP levels of the subgroups of ALB 30–40 g/l and the ALB ≥ 40 g/l in the AA+T2DM group were significantly higher than that of the T2DM group. There were no significant differences in the mean FPG levels in these groups (*P* > 0.05). There were no statistically significant differences in the levels of HbA1C, GA, GSP and FPG between the T2DM group and the healthy group with the ALB levels of 30–40 g/l and the ALB levels of ≥40 g/l (*P* > 0.05) (Table 4).

**Table 4:** Comparisons of blood glucose parameters in the AA group classified by the albumin level (mean ± SD)

Group/ALB (g/l)	*n*	HbA1c (%)	GA (%)	GSP (μmol/l)	FPG (mmol/l)
Healthy control ALB ≥40	114	5.22 ± 0.44	12.60 ± 1.46	171.91 ± 27.97	5.10 ± 0.53
AA Group					
ALB 30‒40	19	5.69 ± 0.48*	11.80 ± 1.64*	169.29 ± 23.01	4.90 ± 0.34
ALB ≥40	21	5.30 ± 0.36	12.67 ± 1.99	196.26 ± 16.74^A^	5.06 ± 0.64
T2DM Group					
ALB 30‒40	29	7.53 ± 1.28	21.74 ± 4.53	250.12 ± 65.22	10.15 ± 5.47
ALB ≥40	59	7.43 ± 1.61	21.61 ± 4.45	261.17 ± 47.43	9.55 ± 3.16
AA+T2DM Group					
ALB <30	8	9.16 ± 2.02*	19.65 ± 1.67*	296.46 ± 51.53*	11.81 ± 5.63
ALB 30‒40	12	8.08 ± 1.57*	22.25 ± 4.63	286.23 ± 39.89*	9.46 ± 4.87
ALB ≥40	10	8.33 ± 1.52*	23.02 ± 5.05	286.83 ± 63.67	9.14 ± 2.73

* *P*<0.05, ^A^*P*<0.001. There were 2 AA patients with ALB < 30 g/l (not shown in the table).

### Correlations between HbA1c and GA in different groups

**Figure 2:**
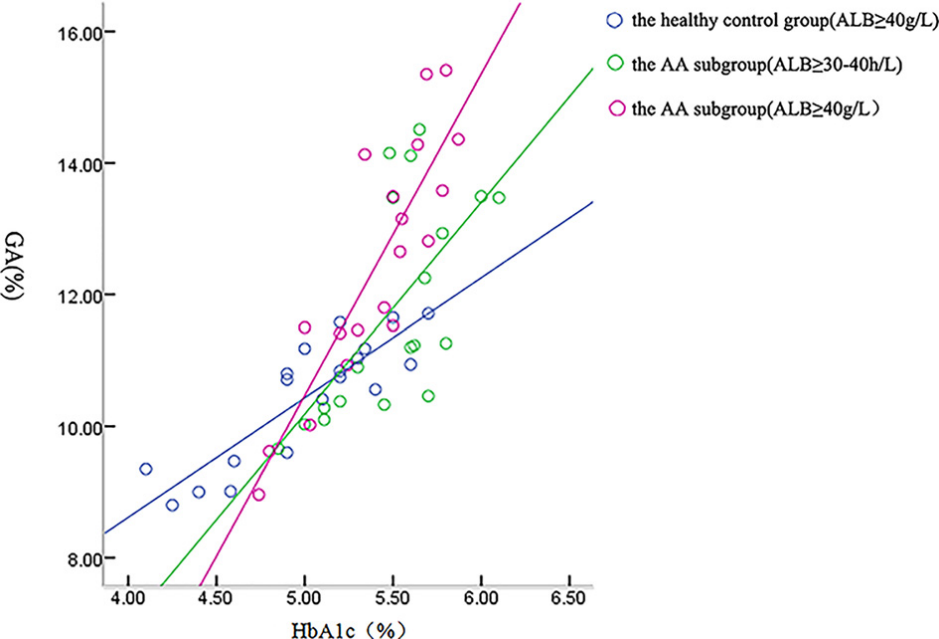
Correlations between HbA1c and GA in the healthy control group and AA subgroup

As shown in Figure 2, there was a correlation between HbA1c in the AA subgroup of ALB 30–40 g/l (*r* = 0.654, *P* = 0.002), which was also observed in the AA subgroup of ALB ≥ 40 g/l (*r* = 0.872, *P* = 0.000) and in the healthy control group (*r* = 0.869, *P* = 0.000).

**Figure 3:**
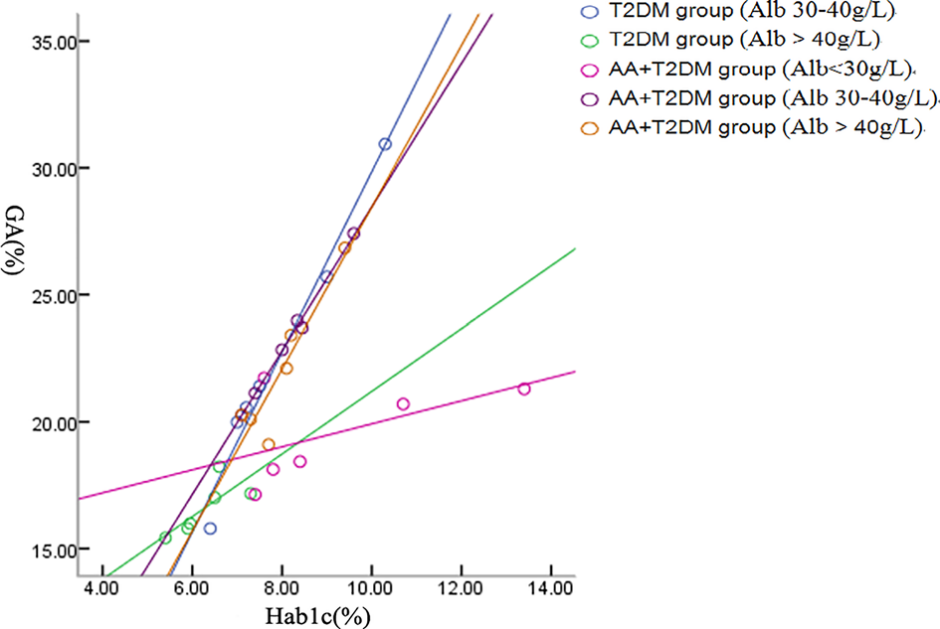
Correlations between HbA1c and GA in T2DM and AA+T2DM groups

Similar correlation analysis was done for the T2DM and AA+T2DM groups. As shown in Figure 3, there was a correlation between HbA1c and GA in the AA+T2DM group (*r*=0.565 for the ALB <30 g/l subgroup, *P* = 0.243; *r*=0.997 for the ALB 30–40 g/l subgroup, *P*=0.000; and *r* = 0.924 for the ALB > 40 g/l subgroup, *P*=0.000). The correlation between HbA1c and GA was also observed in T2DM group (*r* = 0.988, *P* = 0.000 for the ALB 30–40 g/l subgroup and *r*=0.780, *P*=0.000 for the alb > 40 g/l subgroup).

## Discussion

Aplastic anemia is a group of hematopoietic stem cell diseases characterized by bone marrow hematopoietic failure; the etiology is not fully understood but including various physical, chemical and biological causes that result in failure in bone marrow hematopoietic function. The clinical manifestations of AA patients include progressive anemia symptoms and blood transfusion that are often needed in prolonged clinical treatment. Studies have been shown that frequent transfusion therapy can lead to hemosiderosis, and overloaded iron is mainly deposited in the heart and pancreatic islets, causing damage to the islet B cells and resulting in islet dysfunction [17,18]. Nishio et al. have also reported multiple cases of blood transfusion leading to hemochromatosis [19]. Approximately 65% of the patients with pancreatic islet disease caused by hemagglutinin-induced hyperlipidemia manifest multiple diabetic symptoms and signs, such as polyuria, weight loss, elevated blood glucose and urine-sugar positive. Therefore, it is of great clinical significance to strengthen the monitoring of blood glucose level in AA patients.

FPG is one of the most commonly used blood glucose monitoring indicators, reflecting the blood glucose levels. In the present study, we first analyzed the fasting blood glucose (FBG) levels at various time points (0, 1 and 2 months) during the study. Based on the results of the correlation analysis, FBG at the three time points showed a very good correlation among them. Although FPG is considered to be a microglycemia monitor, it can also be used to indicate that the subjects’ blood glucose control was relatively stable during the study period. In the present study, FPG was able to reflect the difference between AA and AA+T2DM. Of note, FPG testing requires a strict coordination with patients; patients need to be fasted for at least 8 h, herefore such compliance is usually poor, susceptible to emotional, stress, diet and other factors, easily leading to false results. Additionally, since AA patients have varying degrees of anemia, strict fasting for 8 h is more likely to lead to dizziness and other symptoms of discomfort. Therefore, we believe that AA patients should not frequently undergo blood tests, and FPG is not a particularly appropriate indicator for long-term blood glucose monitoring among patients with AA, especially in those with Type 2 diabetes.

HbA1c is formed when glucose binds to the N-terminal valine of the β-chain of an Hb molecule. The glycation process is non-enzymatic; therefore, it is only dependent on time, glucose concentration and Hb levels. Red blood cells are generally thought to circulate for approximately 106 days with a variation of ±20% [20] and thus, HbA1c represents the average glucose concentration that Hb is exposed to over a period of approximately 106 days. This integration of glucose exposure over time makes HbA1c a valuable marker in assessing glycemic control over a longer period of time that can be assessed using glucose, which only indicates glycemia at a single time point. The major shortcoming of this test is that it can be affected by the red blood cell life span that varies significantly among individual patients. Published studies on anemia patients, including hemolytic anemia and thalassemia, which have shown that the levels of HbA1c in those patients are mostly low [10,21,22]. However, our results from the present study demonstrated that the patients with AA or AA+T2DM had significantly higher HbA1c values relative to the control group. Further analyses of HbA1c in patients with different Hb levels found that the results of HbA1c testing were not affected in patients with no anemia or mild anemia, whereas the HbA1c levels in the patients with moderate, severe and extremely severe anemia were significantly higher than that in other groups. There was a correlation between the high level of HbA1c and the severity of anemia. Considering the relatively small sample size in the present study, this finding needs to be confirmed in future large-scale studies.

The possible reasons for the elevated HbA1c level in patients with AA observed in the present study are not clear. The following factors may be considered. First, the average erythrocyte half-life in patients with aplastic anemia is about 19.2 (±3.3) days, shorter than that in healthy subjects. The cause of anemia in patients with AA is mainly due to the dysfunction of bone marrow hematopoietic stem cells and/or hematopoietic microenvironment; hematopoietic red pulp is replaced by fat, resulting in reduced whole blood cells. AA patients often have reduced bone marrow hematopoietic function with absolute reduction of reticulocytes, leading to an imbalance between mature red blood cells and young erythrocytes in the peripheral blood. It has been reported that the rate of glycation in young red blood cells is remarkably lower than that of the mature red blood cells [6,7,9]. AA patients have a higher proportion of mature red blood cells in the peripheral blood. Therefore the natural glycation rate is also increased, which may lead to a higher HbA1c level in AA patients. Second, AA patients have progressive anemia, bleeding, and infection (with fever) and most undergo blood transfusion treatment, resulting in a long-term iron overload and deposition in the pancreatic islets, leading to pancreatic islet cells damage, the emergence of insulin resistance,and finally an elevated blood glucose level [18,23]. Third, drug therapies in AA patients may also be an important factor for increasing blood glucose level. Androgen is a common treatment for patients with aplastic anemia, and it has been shown that androgen can reduce or reverse the telomere-shorting effects observed in aplastic anemia, thereby extending cell life. If the mature red blood cells cannot be added, this effect will exaggerate theimbalance in the proportion of young and mature erythrocytes. Diamond et al. have reported that the uptake rate of glucose is significantly reduced after administration of methyltestosterone, demonstrating that testosterone can induce insulin resistance in young healthy women [24]. It has also been suggested that androgen therapy can increase FBG level [25]. Mitsuzuka et al. have reported that, after a 1-year androgen treatment in prostate cancer patients, their mean FBG and glycated hemoglobin levels are increasedby 3.9% and 2.7%, respectively [26]. In addition, it has been pointed out that the commonly used drugs for the treatment of aplastic anemia, such as tacrolimus, cyclosporine A and FK-506, can lead to glucose metabolism disorders [27–29]. The most possible cause may be that these drugs have a direct damage to pancreatic islets, resulting in a reduced insulin secretion. In addition, high concentrations of FK-506 can inhibit the binding of insulin receptor, exacerbating insulin resistance [30]. Thus, we conclude that HbA1c cannot accurately reflect the blood glucose levels in patients with AA or AA+T2DM.

In the present study, we found no significant difference in the GA level among the patient groups and the control group in the overall comparisons (Table 1). These results indicated that GA may not be affected by anemia, which could reflect the blood glucose level. Therefore can be used as a reliable blood glucose monitoring indicator for AA patients with T2DM. It has been suggested that GA is a suitable indicator for blood glucose monitoring in patients undergoing iron-deprivation therapy [10]. It is also applicable to AA patients undergoing both blood transfusion and iron-deprivation therapy.

In the present study, we compared the GA levels among the subgroups of AA+T2DM and T2DM patients grouped according to their ALB levels (Table 4). The mean GA level in the AA subgroup with ALB level of 30–40 g/l was significantly lower than that in the healthy group (*P* < 0.05). However the difference in GA levels between the AA subgroup with ALB level of ≥40 g/l group and the controls was not statistically significant. In the patients with AA+T2DM, the GA level of the subgroup with the ALB < 30 g/l was significantly lower than that in T2DM group (*P* < 0.05). However, but the difference in GA levels between the subgroup with ALB level of 30–40 g/l and the T2DM group was not statistically significant. From the above results which indicated that one of the limitations of GA is that GA is affected by the total albumin level. It is reported that GA is not a good indicator for glucose monitoring in patients with cirrhosis combined with hyperglycemia when their ALB level is <30 g/l. In addition, it has also been suggested that GA is affected by the rate of albumin metabolism, and that GA cannot be used to accurately assess the blood glucose level in patients with nephrotic syndrome, thyroid dysfunction or liver cirrhosis since the structure and half-life of albumin are often affected under those pathological conditions [31,32].

In the present study, we also analyzed the correlations between the GA and HbA1c levels in subgroups of patients classified by different albumin levels. As shown in Figure 2, there was a positive correlation between GA and HbA1c in the AA group and the healthy group, although the correlation in the subgroup with ALB of 30–40 g/l was slightly worse than other subgroups. As shown in Figure 3, although the correlation between GA and HbA1c levels in the subgroup with ALB < 30 g/l was not statistically significant (*r* = 0.565, *P* = 0.243), there were significant correlation between GA and HbA1c levels in other two subgroups with ALB of 30–40 g/l and ≥40 g/l, respectively (*P* = 0.000). Furthermore, it was found that there was a strong correlation between GA and HbA1c (*P* = 0.000) in patients with AA or AA+T2DM. However, GA was not useful in AA patients with ALB levels below 30 g/l and GA was not affected when ALB levels were in the range of 30–40 g/l. Further investigation is needed to confirm the usefulness of GA in monitoring blood glucose in AA patients with T2DM.

In the present study, we also compared the measured values of HbA1c, and HbA1c estimates 1 / 3GA values for the AA patients (Figure 1). The measured HbA1c values were significantly higher than the HbA1c estimates, and the HbA1c estimates were close to 1/3GA values [16]. These results were consistent with the findings reported by Koga et al [10]. Tahara et al*.* [33] have found that GA = 2.74 × HbA1c + 2.26, with HbA1c7.0% corresponding to a GA value of 21.4% in 154 Japanese T2DM patients. The results are similar to that from a study of 939 Chinese diabetic patients that GA = 2.452 × HbA1c + 3.636 with HbA1c7.0% corresponding to be a GA value of 20.8%. Therefore, when blood glucose is relatively stable and HbA1c does not apply to certain conditions such as hemolytic anemia and autoimmune hemolytic anemia, 1/3GA value could be used to estimate HbA1c in the clinical practice.

Glycosylated serum protein (GSP), also known as serum fructosamine (FMN), which is the product (ketamine substance) of non-enzymatic reaction between blood glucose and a variety of proteins in the serum, mainly albumin and can reflect the average blood sugar levels in recent 2–3 weeks. In the present study, the GSP levels in AA patients were higher than that of healthy controls (*P* < 0.05) and the same figure in the AA + T2DM patients was greater than that in T2DM patients (Table 1). However, there were no significant correlations between GSP levels and the Hb levels of (Table 3) or ALB levels (Table 4). The possible reasons were not clear. Whereas it may be associated with many factors, including hemolysis, blood and hyperbilirubinemia [10,34–36]. In addition, the GSP assay contains more ingredients, which can affect the final results due to reaction time and methodological defects. For instance, glycated amino acids and glycated globulin can be mixed with GSP that lead to false results.

Based on the above comparisons on the application values of HbA1c, GA, GSP and FPG, it is clear that GA is an ideal indicator for blood glucose monitoring in patients with AA who could not undergo multiple blood tests. However, GA is not applicable when the AA patients have hypoalbuminemia. Additionally, HbA1c is a reasonable indicator for blood glucose monitoring in AA patients with mild anemia. Nonetheless can overestimate the blood glucose levels in AA patients with moderate and severe anemia. Taken together, the level of ALB and the severity of anemia, alongside with other factors, should be taken into account in the selection of blood glucose monitoring method for AA patients with T2DM.

## Limitations of the study

Our study was limited by the relatively small sample size. Future studies will be needed to recruit larger and more populations. Further functional studies are required.

## Abbreviations

AA: aplastic anemia
ALB: albumin
FBG: fasting blood glucose
FMN: fructosamine
FPG: fasting plasma glucose
GA: glycated albumin
GSP: glycosylated serum protein
Hb: hemoglobin
HbA1c: hemoglobin A1c
IBIL: indirect bilirubin
T2DM: type 2 diabetes mellitus
TBIL: total bilirubin
